# A rare case of massive upper gastrointestinal bleeding caused by Brunner’s gland adenomas combined with a neuroendocrine tumor

**DOI:** 10.1055/a-2876-1858

**Published:** 2026-06-12

**Authors:** Weihong Meng, Yutao Ma, Rui Meng

**Affiliations:** 1 Gastroenterology and Endoscopy Unit 643828 Qilu Hospital of Shandong University Dezhou HospitalDezhouChina

A Brunner adenoma is located in the submucosa of the duodenum, mostly in the proximal duodenum. It is caused by benign hyperplasia of Brunner’s gland cells, with an incidence of less than 0.01%, accounting for approximately 5–10% of benign duodenal tumors. 1​ This lesion most commonly occurs in the duodenal bulb (57.00%), followed by the duodenal papilla (27.00%) and the descending part of the duodenum (7.00%), and may migrate from the duodenal bulb into the gastric antrum. Based on morphological features, it is classified into three types: diffuse nodular hyperplasia type, nodular hyperplasia type, and solitary tumor type (the latter being the most common, including two subtypes: pedunculated and sessile 2​ ​3​ ). The tumor is usually asymptomatic, and a small number of cases may present with upper gastrointestinal bleeding, gastric outlet obstruction, and intussusception. We report a rare case of a 5.0×4.0 cm long‑pedunculated polyp consisting of a Brunner gland adenoma with a component of neuroendocrine tumor, which caused massive upper gastrointestinal bleeding.

We report a case of a young women with a previously healthy status and no history of medication use, who was admitted to the hospital via the emergency department due to abdominal discomfort accompanied by dizziness for 10 hours and melena for 2 hours. The patient had a transient syncope with the loss of consciousness at home, which resolved spontaneously a few minutes later, and she was then taken to our emergency department by her family members. A complete blood count was performed in the emergency department, showing a hemoglobin level of 99.00 g/L; abdominal computed tomography revealed no obvious abnormalities. After symptomatic treatment, the patient was consulted by physicians of our department and subsequently admitted to the ward. During symptomatic treatment after admission, the patient developed melena three more times, with a total volume of approximately 1,000 g and no hematemesis. In the meantime, she experienced two episodes of transient syncope, each resolving spontaneously within 1 to 2 minutes. Symptomatic treatments including blood transfusion and volume expansion were administered, and a recheck of hemoglobin showed a drop to 61 g/L. An emergency endoscopy was then performed to identify the cause of bleeding. Upon advancing the endoscope to the gastric cavity, diffuse adherent black blood clots were observed with no obvious bleeding points. The endoscope was further advanced to the duodenojejunal flexure (bulb and descending duodenum), where a large pedunculated polyp was found ( Fig. 1 ); a red thrombotic head was visible at the root of the pedunculated polyp ( Fig. 2 ), and no other lesions that could cause bleeding were detected. We then decided to resect the responsible lesion. Given the thick root of the polyp, to ensure successful resection and prevent postoperative bleeding, we used a ligation device to tighten and release it around the polyp’s root. A hot snare was then used to perform electrocoagulation and electroresection of the polyp above the ligation device, and three titanium clips were applied to close the wound for the prevention of postoperative bleeding. The procedure was completed smoothly without any bleeding. The patient was subsequently hospitalized for observation for 3 days and discharged with no evidence of active bleeding ( Video 1 ).

**Fig. 1:**
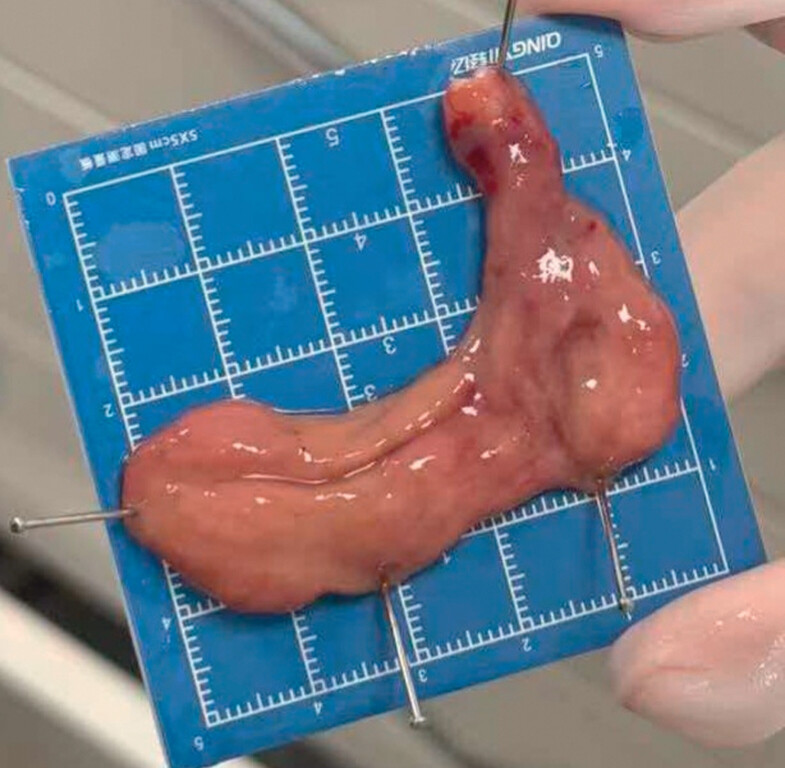
The resected specimen of the 5.0×4.0 cm long-pedunculated polyp in this patient.

**Fig. 2:**
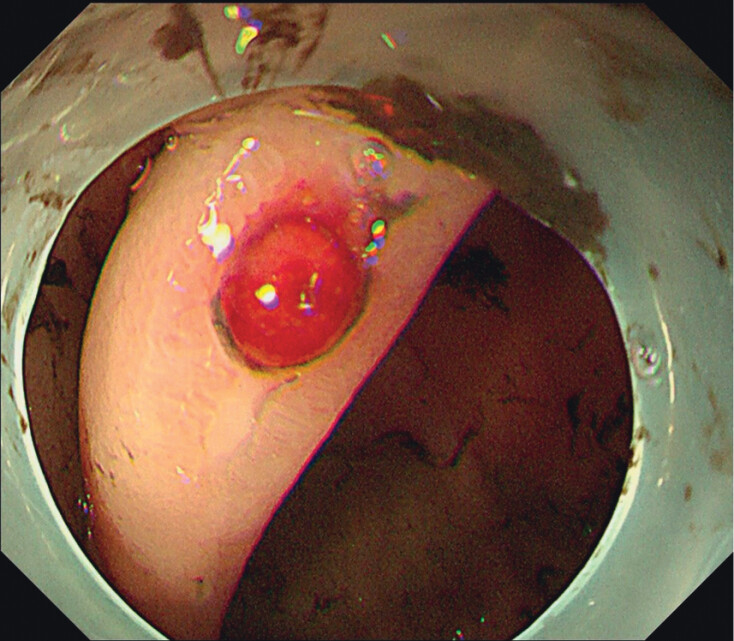
The red thrombotic head at the base of the patient’s polyp.

**Video 1** The procedural process of emergency endoscopic treatment performed on the patient.


Postoperative pathology revealed a Brunner gland adenoma (an elevated lesion in the descending duodenum) with multifocal neuroendocrine cell hyperplasia and focal formation of a neuroendocrine tumor (NET, G1). The maximum diameter measured microscopically was approximately 0.4 cm, with negative resection margins ( **Figs.** 3 **–** 5 ). Duodenal Brunner’s gland adenomas are predominantly benign lesions that generally require no specific treatment. However, endoscopic or surgical resection should be considered when the tumor is large or causes adverse events such as bleeding. 4​ The recurrence rate after endoscopic or surgical treatment is low, and the prognosis is favorable. 5​ This case report presents a giant Brunner adenoma combined with a neuroendocrine tumor. It is considered that the large tumor mass caused prolonged traction and friction on the stalk, resulting in ulceration and bleeding at the polyp stalk, which led to massive gastrointestinal hemorrhage and the transient loss of consciousness in the patient. The results show that endoscopic-assisted pre-ligation resection is an effective method for the treatment of giant duodenal Brunner’s adenomas. Considering that the patient’s neuroendocrine tumor is at the G1 stage, endoscopic resection is also suitable, achieving excellent clinical outcomes.

**Fig. 3:**
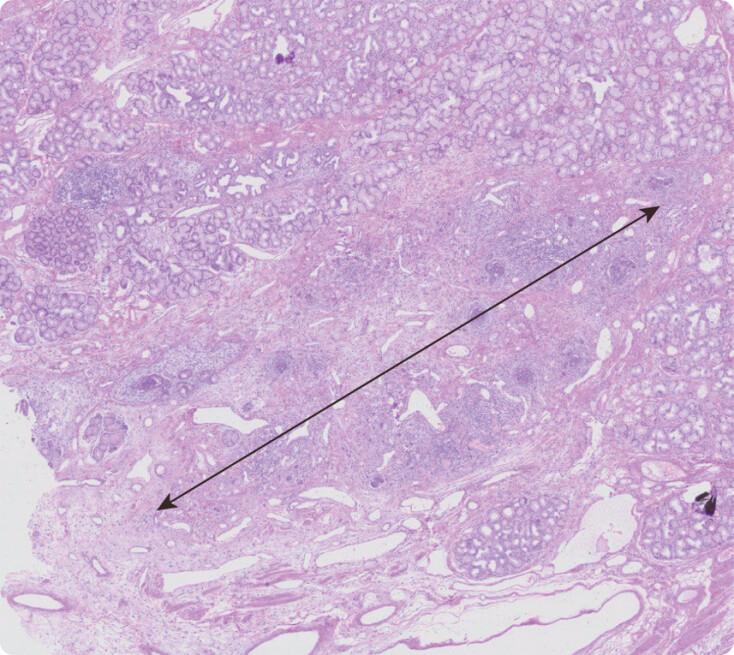
The area of the neuroendocrine tumor indicated by the arrow has a maximum diameter of approximately 0.4 cm.

**Fig. 4:**
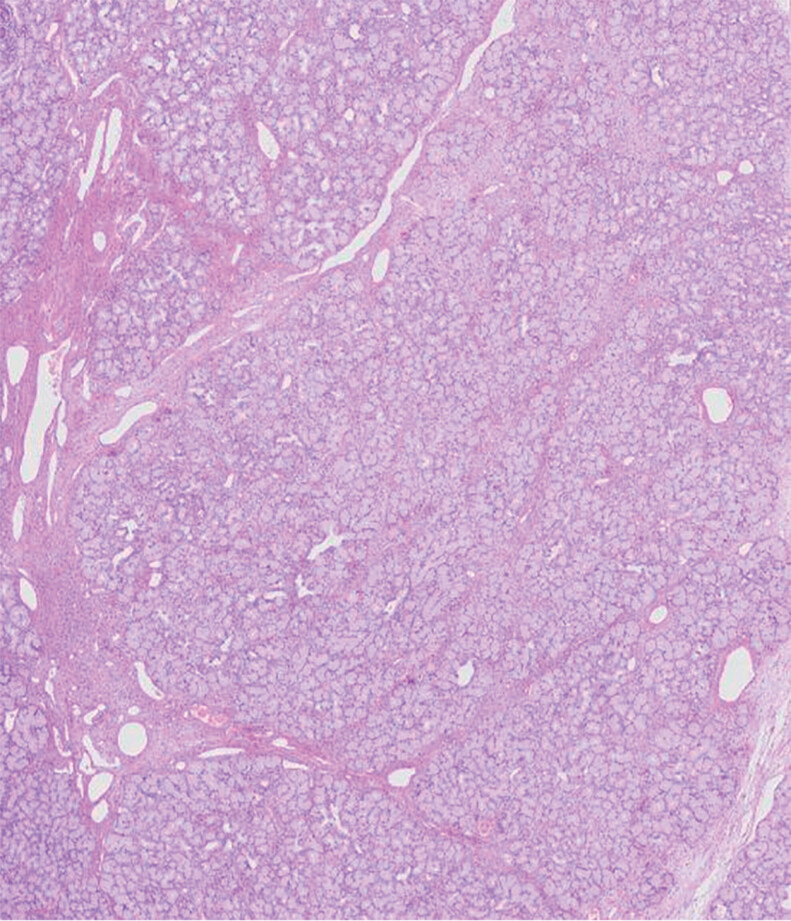
This image shows Brunner’s glands.

**Fig. 5:**
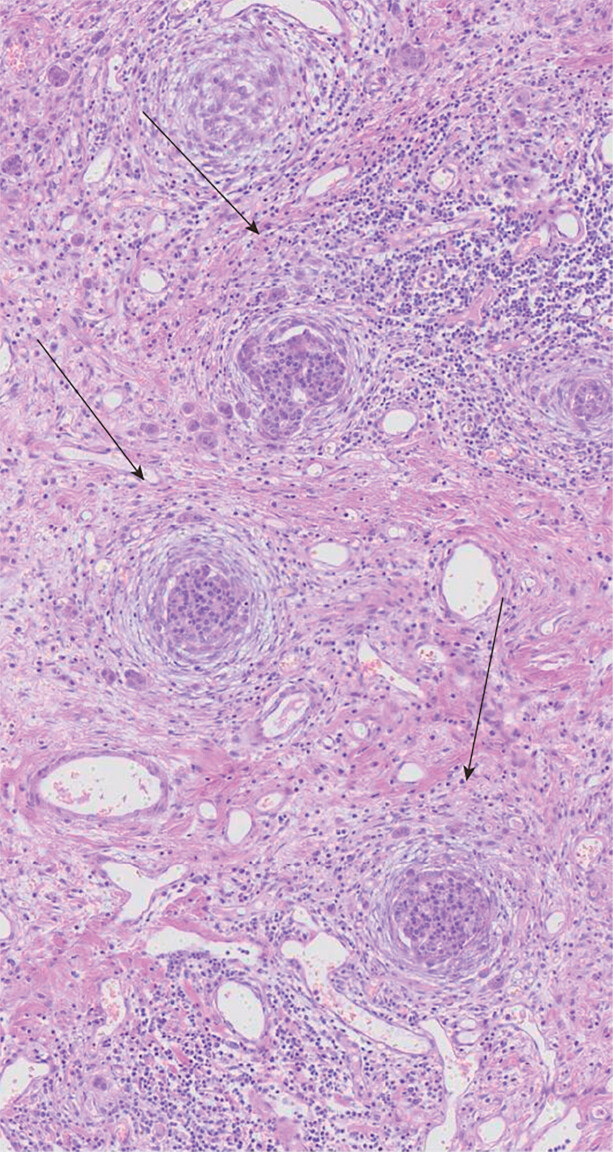
→The arrow indicates neuroendocrine cells.

Endoscopy_UCTN_CODE_CCL_1AB_2AC_3AG

## References

[1] Chang C W Chang W H Shih S C Lin S C Yang T L Wang T E Proboscis-like Brunner’s gland hyperplasia Am J Surg 2008 196 e33 e34 10.1016/j.amjsurg.2007.10.025 18585675

[2] Walden D T Marcon N E Endoscopic injection and polypectomy for bleed-ing Brunner’s gland hamartoma: Case report and expanded literature re-view Gastrointest Endosc 1998 47 403 407 10.1016/s0016-5107(98)70228-7 9609436

[3] Patel N D Levy A D Mehrotra A K Sobin L H Brunner’s gland hyperplasia and hamartoma: imaging features with clinicopathologic correlation AJR Am J Roentgenol 2006 187 715 722 10.2214/AJR.05.0564 16928936

[4] Iwamuro M Kobayashi S Ohara N Adenocarcinoma in situ arising from Brunner’s land treated by endoscopic mucosal resection Case Rep Gastrointest Med 2017 2017 7.916976E6 10.1155/2017/7916976 PMC5420418 28512587

[5] Dhali A Ray S Ghosh R Massive upper gastrointestinal hemorrhage in Brunner’s gland hamartoma of duodenum Cureus 2021 13 e15875 34336406 10.7759/cureus.15875 PMC8312786

